# Mr. Cuming, on Gun-Shot Wounds

**Published:** 1804-05-01

**Authors:** Ralph Cuming

**Affiliations:** Romsey


*April*, 1804.

---

### *To the Editors of the Medical and Phyfical Journal.*

GENTLEMEN,

THE treatment of gun-shot wounds is a subject of great importance, and well merits the serious attention of the Faculty; and in a peculiar manner that of the younger branches of the profession, who intend dedicating their early years to the service of their country, which during war never fails to present a wide field for improvement, whether their views are directed to the navy or army. The deprivation and sacrifices of numberless comforts will be fully compensated by the opportunities they will have, of giving the finishing stroke to their education, if extensive experience can add any thing to therapeutic and prophylactic skill. It is greatly to be lamented that most of the authors who have written on this subject, never saw a shot fired in anger; and from their want of personal experience, are very inadequate to the task they have assumed, of giving lessons of instruction on topics the most serious and important, for such they must ever be considered, where the lives of British heroes are in the hands of men whose ideas of treatment have been borrowed from those productions. Would not the tear of sorrow, and the heart rending sigh, burst forth, if we were told that a Wolfe or an Abercrombie was lost to his country from maltreatment? Then, how careful the student ought to be in his choice choice of authors. I am of opinion that Mr. John Bell's discourses on the treatment and nature of wounds should form a part of every Surgeon's library; he writes like a man who has been engaged in scenes of war, or one whose hospital practice has enabled him to treat the subject in a masterly manner. I have been informed that he derived a considerable part of his knowledge from visiting the ships under the command of Lord Duncan, subsequent to the victory he obtained over the Dutch fleet; but on examining the title page of his book, I find it was published before that action took place: however, his visiting the wounded is a proof that he has been assiduous in obtaining information on the subject.

A few cursory remarks are all I shall presume to offer, in my endeavours to point out to the young surgeon, that plan of treatment which experience teaches is most likely to crown his endeavours with success. To think of laying down rules which are never to be deviated from, would be the most consummate folly and presumption; for much must ever be left to the ingenuity and prompt decision of the surgeon. If not, from whence would our most noble improvements proceed?

The wounds in a naval engagement are generally from cannon shot, and consequently much more dreadful than those of the musket; and often in proportion to the extent of the wound may the danger be estimated. The men who nearly compose the aggregate of a ship's company, are from their peculiar modes and habits of life, such as cannot boast of the best of constitutions; and many of them are tainted with the scurvy, a disease which formerly made great havock in the service; but the liberality of government has for years provided a remedy (lemon juice) which has nearly eradicated this scourge of sailors.

Then the treatment of a wounded seaman is briefly this, which may in almost every case be equally applicable to a soldier. In flesh wounds, or where there is only a loss of muscular substance, the wound must be well cleaned with a sponge moistened with water; the hæmorrhage stopped, if issuing from the mouth of a large vessel; and the tourniquet must be applied and properly managed, until the vessel is taken up, which is to be done by means of the tenaculum or forceps, taking care not to include any of the cellular membrane with which it is surrounded. If it cannot be secured in this manner, a needle must be passed round it, including as little of the surrounding parts as possible. Your success in saving the life of your

 patient, patient, and performing a speedy cure, will greatly depend upon your dexterity in securing the vessels, which I shall elucidate by relating to you the case of a man, wounded at the battle of Copenhagen, who was placed under my care at Yarmouth hospital, together with nearly the whole of those who had lost their limbs. This person had his arm amputated at the insertion of the deltoid muscle; the operation appeared to have been performed in a very proper manner, so much so, that the face of the stump was perfectly covered with the integuments, and at the time he was received it was nearly cicatrized. That part where the brachial artery hung from being still open, proved a source of great distress to the patient, for every time it was touched, with a view of disengaging it, the sensation arising from a slight pull, somewhat resembled an electrical shock; which clearly evinced that a nerve had been included in the ligature: and it nearly cost the patient his life, for violent inflammation took place, pervading the whole of the stump, extending itself to the axilla, breast, and back. The parts were wrapped up with emollient poultices; and by the aid of fomentations, it terminated in suppuration, so extensive, that had he not been well supported with beef tea, wine, porter, decoct. cinchonæ, &c. he must have sunk under it.

It is surprising how this man escaped that fatal complaint, locked jaw; but so various and inexplicable are the springs which actuate Nature, that the prognosis of the most skilful is often erroneous.

Should the bleeding flow from every point of the wound, you may suspect that your patient is of a scorbutic diathesis; that the crassamentum of the blood is broke down by this or some other disease. In this case you are to cover the wound with lint, and by graduated tow compresses and rollers secure it; taking care not to remove the dressings until it begins to suppurate. In such a case, the inflammation will be purely local, and a generous regimen may be resorted to immediately; for in these cases, where there is a great loss of substance, the discharge is always profuse; and if your patient's strength is not kept up by strong soups, jellies, and such things as will restore lost stamina, he will sink into a low nervous fever, and die.

Here I must caution you against giving the bark in substance, for those who have been so profuse in their encomiums on this *drug*, in this state, have much to answer for. If the surgeon is not blinded by prejudice, he will discover his patient losing his appetite, and becoming more and and more debilitated every hour, whilst he is pouring in large libations of this *ne plus ultra* medicine. Believe me, more is to be done by diet. In these cases, make up your mind never to give it except in decoction; a little of the tincture may be added, and the mixture acidulated with acid. vitriol. Such medicine, given in moderation, will assist the digestive powers, but the bark in substance clogs up the stomach, checks the secretion of the gastric juice, and totally unhinges it, rendering all its efforts to convert the food into chyme useless and abortive. The stomach is an organ endued with such sensibility, that it always sympathizes with the injuries which the body sustains; and to give bark in substance, under such circumstances, is productive of a train of symptoms the most distressing, viz. pyrexia, vomiting, &c. Such symptoms may occasionally arise from the injury itself; but I am fully convinced they are most frequently the effects of this highly irritating and nauseous drug.

In flesh wounds bleeding is seldom necessary, and is generally productive of the worst of evils, for it often happens that men in action are quite exhausted, from loss of blood, before the surgeon can attend to them; a well directed broadside makes so much havock, that a third of the wounded die from want of medical assistance. Allowing that a man should lose very little blood before his wounds were secured, if they were extensive the discharge from them during the cure would reduce him too much, even admitting that every thing comfortable and nourishing was at his command, which is not always the case; so you perceive, how absolutely requisite it will be for you to guard against bleeding, or any other evacuation that may tend to debilitate your patient, for local inflammation will generally yield to local applications, such as emollient poultices and fomentations; and in these applications great judgment is requisite to know the precise point where to discontinue them. It frequently happens that the skin takes on a diseased action, assuming an erysipelatous appearance, at the time the wound is discharging very profusely; in such a case, cold applications to the surrounding inflammation will be proper, namely, a weak solution of cerussa acetata, renewing rags dipped in it as often as they grow warm. By this means the temperature of the part will be reduced, and the patient relieved from considerable pain, the natural concomitant of inflammation.

The best diet, when the strength is sinking under profuse gleetings, is animal food, in the shape of jellies. As the stomach should be distressed as little as possible by the process of digestion, if sufficient nutriment cannot be conveyed into the system in this manner, it may be thrown into the intestines per anum, with a few drops of laudanum. Many a valuable life has been saved in this manner. When the granulations have arrived at the point when bleeding and fungous flesh make their appearance, the pressure of the roller and compresses should be increased, which will greatly facilitate the healing process, and enable you to cicatrize the wound, without having recourse to escharotics, so frequently used by the surgeons of the old school.

With respect to wounds of the hands, feet, and limbs, accompanied with fracture and splintered bones, it is highly requisite for you to be possessed of intrepidity, tempered with coolness; for what you decide upon, must be exerted without delay, and you will be careful never to amputate without circumstances render it absolutely necessary; every surgeon possessed of anatomical knowledge, can quickly determine. If you find the nerves and tendons injured, the muscles much lacerated, and spicula drove into the very heart of a limb, with or without much hæmorrhage, in such a case amputate immediately; and here permit me to remark, your fore finger will make the best probe, for you must use it to ascertain with precision the extent of the injury.

In wounds of less magnitude, when there is a probability of saving the limb, by using your fore finger and thumb, you will be enabled to pick away every particle of bone, for the smallest atom, if felt, must not be left behind, and you must recollect that this is your only time for cleansing the wound from every foreign body; to leave any thing unfinished now, and attempt it when inflammation and tension had taken place, would be extremely cruel, and subject your patient to inexpressible torture. It will be frequently necessary for you to use a knife in detaching splinters, and if you cannot disengage them without enlarging the wound, do it boldly; it is better that you should risk dividing an artery, than to lose your patient by trismus, which is often occasioned through the irritation of splinters

Wounds of this description often mortify; you may say, if there is any likelihood of such an occurrence, it will be most prudent always to amputate. Such reasoning is erroneous; it is a common thing for stumps to become sphacelous, an evil originating sometimes from crowding the the wounded together, and frequently from a cachectic state of the body, independent of external causes; therefore, when this occurs, little judgment will determine, whether a small or large surface is most likely to terminate favorably; and in this dilemma, it affords me infinite pleasure to be enabled to direct you to the choice of a medicine which merits the highest encomiums, namely, nitre, finely pulverized, sprinkled all over the part, completely covering it at every dressing, which will as surely stop its progress as mercury will that of the lues venerea; and if the fœtor is ever so disagreeable, it will, in the space of twelve hours, entirely subdue it. You must not neglect to wash the part with vinegar and decoct. cinchonæ, administering the latter internally with port wine, supporting the strength with strong jellies, as directed above.

When a limb is carried away with a shot, or splinter, you must go through the regular plan of amputation, performing your operation at a distance from the wound, to leave integuments for covering the face of the stump. If the limb is too much shortened to admit of amputation, the flap operation of the hip or shoulder must be performed. A marine, who had his arm blown off with a shot at the battle of Copenhagen, near the shoulder, was placed under my care at the naval hospital in Yarmouth. The surgeon of the ship he belonged to, having many men wounded, and being in want of assistants, (which I am sorry to say is too often the case in the Navy) was constrained to content himself by securing the vessels, and dressing it up in the state he received him in the cockpit. It soon became, from the retraction of the muscles and integuments, a complete sugar loaf stump, and his cure bore some resemblance to a resurrection, for he had to contend with every dangerous and unpleasant symptom; he was reduced to a mere skeleton by the discharge.— During the cure he was seized with tetanus and trismus, and laboured under these complaints longer than ever I knew any man in such a state. The wound was dresed with lint dipped in laudanum; opium and æther were prescribed liberally, and the cure perfected by decoct. cinchonæ, wine, nourishing jellies, &c. but not until an exfoliation of nearly the whole of the remaining bone came away. It is worthy of remark that, generally speaking, all the operations of the surgeons in that action were well performed, though in a cockpit with candle-light, and would have done honour to any one on shore, with the advantage of plenty of assistants and day-light, unannoyed with the cries of the wounded, din of battle, and roaring of cannon.

But there was one exception; and this old surgeon, being probably nervous, forgetting to keep an eye on the edge of his knife, and not saving a sufficiency of integuments, some of his patients were detained in the hospital, three, four, or five months, on account of exfoliations. When there is not a sufficient covering left for the bone, the cure will be greatly expedited by using tight rollers, which prevent retraction.

The appearance of the countenance will greatly assist you in forming your prognosis; if the patient is of a florid, clear, and healthy appearance, you may augur favorably; but if he is of a swarthy, sallow, cadaverous aspect, your hopes will not be animating; such patients will bear a greater proportion of wine than the former, for whose recovery ale or porter will be most conducive. When circumstances will admit of it, take care to keep the wounded at a considerable distance from each other, and if any of the stumps or wounds look flabby or ill conditioned, do your utmost to obtain their removal, and total separation from the rest; the poisonous effluvia emanating from a single patient may infect the whole, therefore the good of the poor sufferers, as well as a regard for your own reputation, will stimulate you to exert every nerve in their behalf.

Fumigating the apartments of the wounded, wherever there are a number collected together, with nitrous vapours, according to Dr. Carmichael Smyth's plan, is of infinite service in correcting fœtor, and subduing contagion.

Wounds of the thorax and abdomen require a very different treatment, the antiphlogistic regimen must be strictly observed; you are to use the lancet freely, according to the urgency of the symptoms. Though these wounds often prove fatal, you are never to despair of success; many instances of recovery from balls perforating the lungs can be adduced, some of which I have witnessed myself. I have often thought that the tinct. digitalis, a medicine which stands high in my estimation, might in some measure supersede the necessity of bleeding so profusely; if given in sufficient quantities, it will check the impetus of the blood, by lessening arterial action, and thereby give the ruptured vessels and lungs an opportunity of healing, without impoverishing the body so much as is generally done. I have experienced its good effects in pulmonary inflammations, arising from other causes, and doubt doubt not of its efficacy in cases where similar symptoms originate from wounds.

Wounds of the joints are always dangerous, and when the capsular ligament is perforated, and the heads of the bones fractured or otherwise injured, the safest plan will be to amputate. The loss of a limb must ever be considered a great deprivation, and when the injury received does not appear very extensive, the entreaties of the patient prevail on the surgeon to attempt the cure; the latter sympathizes with him, and from a supposition that there is a chance of a favorable issue, he remains passive, but seldom has the happiness of bringing him off, even with an anchylosed joint. Bleeding has been carried to great lengths in such cases, and I believe great mischief has been done by erring on what may be considered the safe side, for local inflammation will be more readily subdued by means of leeches, and the cold saturnine lotion. How often do we perceive marks of inflammation, when the pulse is weak and feeble, when the flesh is wasted, and strength exhausted? Much more might be said, but I am afraid I have encroached too much on the limits of your publication. I shall conclude by craving your indulgence for the insertion of the following remarks.

No class of men whatever have rendered their country more essential benefit than naval surgeons; yet strange to tell, they are not remunerated for their services, as well as their brethren in the army. This is a circumstance not known to the bulk of the nation, but it is too true; and it is to be hoped that a noble Lord will exert himself as much in their behalf, as a noble Duke has done for those in the army; very little addition has been made to their emoluments since the days of Queen Anne! This is a subject of more national import than may be imagined at first sight. The reason why the army is better supplied with surgeons than the navy, is very obvious; their pay and treatment is better, and when the nature of their respective services is duly estimated, taking into consideration the deprivations and sufferings of both parties, there can be no difficulty in deciding which of them ought to receive the most ample and handsome compensation. Every admiral and captain in the service last war, had to lament the want of medical men; and it is well known that many a brave fellow (whose useful life might have been saved to his country) bled to death from the same cause. The modesty and forbearance of the medical department in the navy, have operated to their disadvantage, otherwise their complaints plaints would have become the subject of parliamentary discussion; and as there are naval characters in both houses, who know how to appreciate the merits of naval surgeons, it is to be hoped they will soon receive merces laborum. I am, &c.

